# Supplementary material for: Identifying trends in reporting on the ethical treatment of insects in research
Source: PLoS One. 2025 Aug 18;20(8):e0328931. doi: 10.1371/journal.pone.0328931 (PMC12360591; doi:10.1371/journal.pone.0328931)
Supplement: S1 Table — Some methods were not explicit even on reading methods sections; some animals were “sacrificed” before further experimentation but the exact methods of sacrifice were not stated. (DOCX) [file pone.0328931.s001.docx]

| Inferred sacrifice method | n |
| --- | --- |
| dehydrated | 1 |
| exsanguinated | 1 |
| proteomics | 1 |
| respirometry | 1 |
| chemical compound analysis | 2 |
| protein extraction | 2 |
| supplement only | 2 |
| dissected | 3 |
| weighed | 3 |
| calcium imaging | 5 |
| euthanised | 5 |
| morphometrics | 5 |
| survival rate | 6 |
| misc. manipulation | 7 |
| nutrient content | 7 |
| trap | 13 |
| immunohistochemistry | 18 |
| microscopy | 20 |
| collected | 27 |
| electrophysiology | 28 |
| nucleic acid extraction | 119 |

**Supplemental Table 1**. Experimental methods that would result in insect death but were left explicitly unstated within the sampled paper, but could be inferred from reading the methods section. Some methods were not explicit even on reading methods sections; some animals were “sacrificed” before further experimentation but the exact methods of sacrifice were not stated.
