# Supplementary material for: Identifying trends in reporting on the ethical treatment of insects in research
Source: PLoS One. 2025 Aug 18;20(8):e0328931. doi: 10.1371/journal.pone.0328931 (PMC12360591; doi:10.1371/journal.pone.0328931)
Supplement: S2 Table — Not stated category refers to those papers with dissection as the method of sacrifice but no reported anaesthetic or analgesic information. (DOCX) [file pone.0328931.s002.docx]

**Supplemental Table 2.** Anesthetic methods employed prior to dissection (n = 302 papers).

Not stated category refers to those papers with dissection as the method of sacrifice but no reported anesthetic or analgesic information.

| Anesthetic method | n |
| --- | --- |
| nitrogen | 1 |
| freezing | 2 |
| unspecified method | 2 |
| CO2 | 18 |
| chilling | 31 |
| not stated | 248 |
