# Supplementary material for: Identifying trends in reporting on the ethical treatment of insects in research
Source: PLoS One. 2025 Aug 18;20(8):e0328931. doi: 10.1371/journal.pone.0328931 (PMC12360591; doi:10.1371/journal.pone.0328931)
Supplement: S1 Fig — Diptera, Hymenoptera, Coleoptera, Lepidoptera, Hemiptera, Orthoptera, and Blattodea were the most represented orders in our analysis. Insecta accounts for papers that reported use of more than 5 orders of insects. (DOCX) [file pone.0328931.s003.docx]

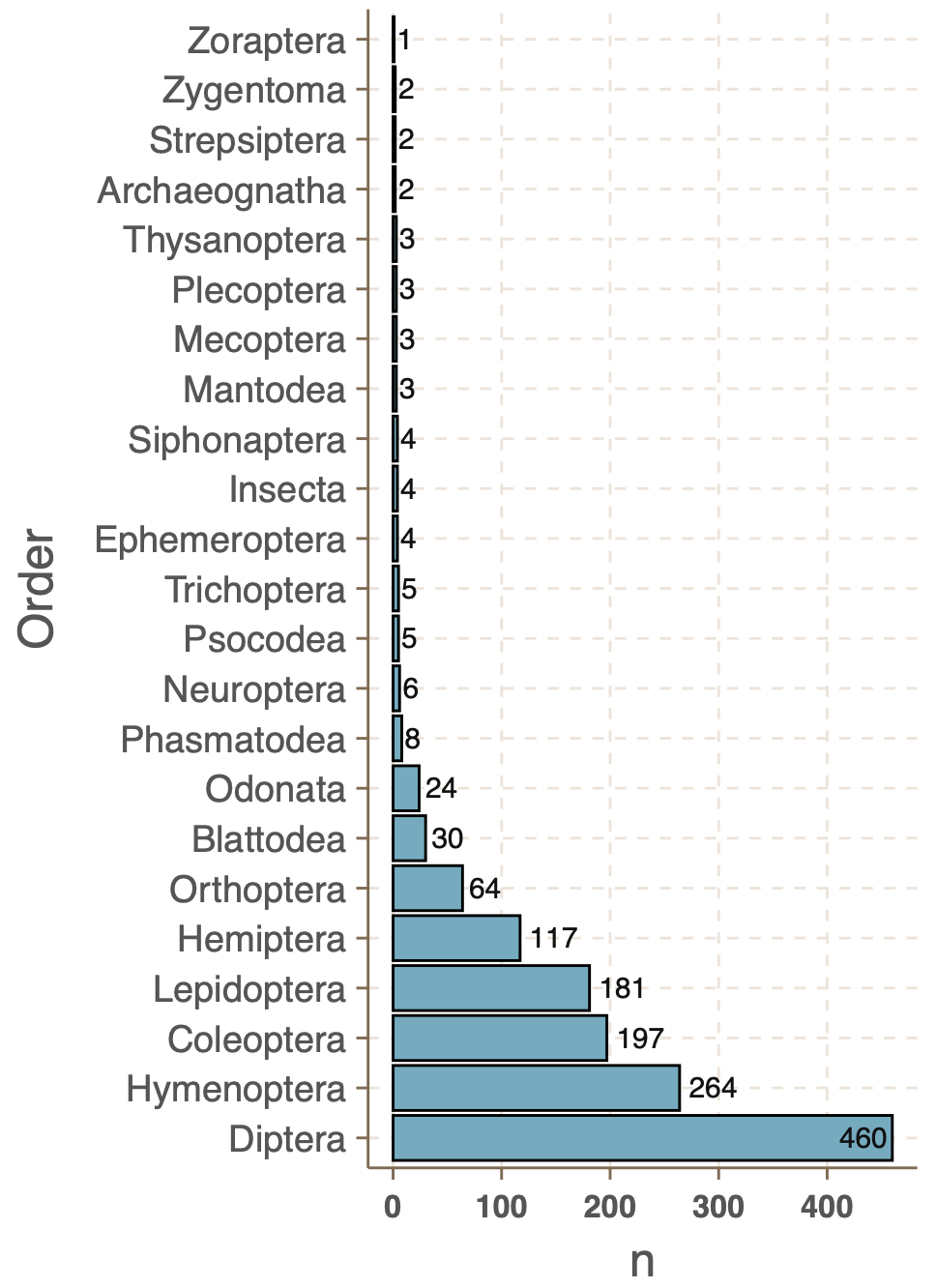


**Supplemental Figure 1. Number of papers that focused on insects of each order.** Diptera, Hymenoptera, Coleoptera, Lepidoptera, Hemiptera, Orthoptera, and Blattodea were the most represented orders in our analysis. Insecta accounts for papers that reported use of more than 5 orders of insects.
