# Supplementary material for: Identifying trends in reporting on the ethical treatment of insects in research
Source: PLoS One. 2025 Aug 18;20(8):e0328931. doi: 10.1371/journal.pone.0328931 (PMC12360591; doi:10.1371/journal.pone.0328931)
Supplement: Attachment — Submitted filename: Research evaluation 24-41322.docx.pdf [file pone.0328931.s005.pdf]

Research evaluation

Trends in Reporting on the Ethical Treatment of Insects in Research

Research Summary

The study analyzes trends in reporting ethical practices in insect research over 20 years (2003–2022) across 15 high-impact journals. It focuses on three key aspects:

1. Reduction of insect use (e.g., statistical power analyses).

2. Refinement of practices (e.g., anesthesia/analgesia use).

3. Transparency in sacrifice methods.

Key Findings

- No reporting on reduction: None of the 1,359 sampled papers mentioned methods to minimize insect use (e.g., power analyses).

- Low anesthesia reporting: 84% of papers involving invasive procedures or sacrifice did not report anesthesia use.

- Increased sacrifice reporting: The proportion of papers mentioning insect death rose from 63% (2003) to 82% (2022).

- Limited ethics statements: Ethics statements increased from 0% to 8%, driven largely by Animal Behaviour, the only journal requiring such statements for invertebrates.

Abstract Evaluation

1. Accuracy in Presenting Results:

- The abstract accurately highlights key findings:

- No reporting of animal reduction methods (0% of papers).

- Increased sacrifice reporting (63% to 82%).

- Low anesthesia reporting (84% of papers omitted it).

- Modest rise in ethics statements (0% to 8%).

- Core statistics (e.g., sample size: 1,359 papers) are clearly stated.

2. Comprehensiveness:

- The abstract covers the main objectives (analyzing ethical reporting trends) and links them to:

- Reproducibility concerns.

- Public trust issues.

- Recommendations for practice improvement.

- Missing nuances:

- Specific impacts of ineffective sacrifice methods (e.g., unanesthetized dissection) on scientific outcomes.

- Disparities between journals (e.g., Animal Behaviour vs. others).

3. Clarity and Focus:

- Concise language, but some phrases need refinement, e.g.:

- "Increase in papers reporting insect death" → Clarify whether this includes direct/indirect sacrifice.

- "Recommendations to improve practices" → Provide an example (e.g., using approved anesthetics).

4. Improvement Suggestions:

- Add a sentence summarizing policy implications (e.g., "These findings urge journals to adopt mandatory ethical reporting guidelines").

- Mention cultural barriers to ethical adoption (e.g., lack of training among entomologists).

The abstract accurately summarizes core findings but needs:

- Clarification of critical details (e.g., journal disparities).

- Stronger links to actionable policy recommendations.

Evaluation of the Introduction

1. Scientific Context Accuracy:

- The introduction effectively frames the importance of ethics in research, highlighting unique challenges in entomology (e.g., lack of legislation).

- Citations of recent studies (e.g., Brunt et al., 2022; Gibbons et al., 2022) support claims about shifting public opinion and scientific interest in insect welfare.

2. Logical Coherence:

- A clear flow from general animal ethics to insect-specific issues, then linking to scientific reproducibility.

- The connection between ethical reporting and research quality (e.g., anesthesia effects on behavior) is compelling and well-supported with examples.

3. Potential Gaps:

- Some citations use indirect links (e.g., Zotero/Google Docs) instead of DOIs or journal pages, reducing credibility.

- The claim that "the public supports ethical oversight for insects" needs stronger backing with survey data (e.g., Drinkwater et al., 2019).

4. Improvement Suggestions:

- Replace informal links with direct references to primary sources (DOIs/journal URLs).

- Clarify the degree of scientific consensus on insect sentience to avoid overgeneralization.

The introduction effectively contextualizes the research gap but requires:

- Strengthening citations with primary sources.

- Clarifying evidence for public opinion claims.

The introduction is strong in building the scientific context and highlighting the research gap, but it needs:

- Enhancing categories with primary sources.

Explain the evidence supporting the allegations related to public opinion.

Summary of Methods

1. Journal Selection:

- 15 high-impact journals (9 insect-focused, 6 general biology journals publishing vertebrate and insect research) were selected based on Scopus CiteScore rankings.

- Journals focusing on non-insect taxa were excluded.

2. Article Sampling:

- 5 random articles per journal per year (2003–2022) were sampled (total = 1,359).

- Non-primary research articles or studies not using live insects were excluded.

3. Data Collection:

- Methods sections were manually screened for:

  - Invasive procedures (e.g., dissection).

  - Sacrifice methods.

  - Anesthesia/analgesia use.

- Automated keyword searches (e.g., "anesth", "CO2") identified relevant terms, followed by manual verification.

4. Statistical Analysis:

- Data were analyzed using R (linear regression, t-tests, ANOVA).

- Trends over time, journal-specific practices, and taxonomic differences were assessed.

The methodology reflects a systematic effort to analyze ethical trends across two decades, with a focus on transparency and repetition in insect research.

Summary of Results

1. No Reporting on Reduction:

- None of the 1,359 papers mentioned statistical methods (e.g., power analyses) to minimize insect use.

2. Low Anesthesia Reporting:

- 84% of papers involving invasive procedures or sacrifice did not report anesthesia/analgesia use.

- Most common methods: chilling/freezing (59%), despite being criticized by veterinarians as ineffective.

3. Increased Sacrifice Reporting:

- Papers mentioning insect death rose from 63% (2003) to 82% (2022).

- 26% of studies used dissection for sacrifice, with 83% performing it without anesthesia ("vivisection").

4. Limited Ethics Statements:

- Ethics statements increased from 0% (2003) to 8% (2022), driven by Animal Behaviour (the only journal mandating ethics statements for invertebrates).

5. Journal Disparities:

- Journals publishing vertebrate research reported anesthesia/sacrifice methods more transparently than insect-focused journals.

These results show a large gap in reporting ethical practices, which threatens the frequency of research and the confidence of the public, especially in light of the wide use of unconventional methods (such as live anatomy).

Summary of Discussion

1. Transparency and Trust Issues:

   - Poor reporting of anesthesia/sacrifice methods undermines reproducibility and public trust, exacerbated by widespread use of ineffective (e.g., chilling) or inhumane practices (e.g., unanesthetized vivisection).

2. Journal Policy Impact:

   - The rise in ethics statements was driven by Animal Behaviour (mandating ethics reporting), yet 70% of its post-2004 papers still lacked statements, highlighting weak enforcement.

3. Cultural and Training Barriers:

   - Entomologists' lack of training in animal welfare principles and absence of legal oversight hinder ethical reform.

4. Precautionary Approach Urgency:

   - Even skeptics of insect sentience advocate precautionary measures (e.g., neural inactivation before painful procedures) to mitigate risks.

5. Practical Recommendations:

   - Collaborate with veterinarians to adopt approved anesthesia methods.

   - Provide training resources and standardized guidelines (e.g., checklists) for transparent reporting.

   - Encourage journals to enforce ethical reporting policies while supporting authors during transitions.

The discussion highlights the need for an institutional and collective transformation in the culture of research to keep pace with moral and scientific concerns, with a focus on education and Cooperation as a park for change.

References Well Attributed, Latest Reference 2024.

References Evaluation

1. In-Text Citation Accuracy:

- Most in-text citations (e.g., Sert et al., 2020; Brunt et al., 2022) correctly match the references list.

- Some hyperlinks direct to non-standard platforms (e.g., Zotero/Google Docs) instead of original sources, reducing credibility (e.g., [(Freelance, 2019)](https://www.zotero.org/google-docs/?DINlI0)).

2. Formatting Inconsistencies:

- Mixed use of "&" and "and" between authors (e.g., Fischer & Larson vs. Crump et al., 2023 and Cuff et al., 2023).

- Inconsistent italics for journal names (e.g., Animal Behaviour vs. Proceedings of the Royal Society B).

3. Link and Source Issues:

- References like Andrews et al. (2024) lack journal/DOI details, relying on non-standard URLs.

- Some references cite preprints (e.g., OSF) without peer-reviewed publication confirmation.

4. Improvement Suggestions:

- Standardize formatting using APA or Chicago style.

- Replace third-party links with DOIs or journal page URLs.

- Add missing details (e.g., volume, issue numbers) for incomplete references.

The references are generally accurate but require:

- Formatting standardization.

- Fixing broken/non-standard links.

- Enhanced transparency through complete source details.

Discussion Highlights

- Transparency gaps: Lack of anesthesia/sacrifice reporting undermines reproducibility and raises ethical concerns (e.g., unanesthetized vivisection in 83% of studies).

- Journal disparities: Journals publishing vertebrate research showed better ethical reporting than insect-focused journals.

- Cultural barriers: Entomologists lack training and guidelines for ethical insect welfare practices.

Core Recommendations

1. Enhance transparency in ethical reporting to improve public trust and reproducibility.

2. Develop standardized guidelines for anesthesia, sample size estimation, and sacrifice methods.

3. Foster collaboration between entomologists and veterinarians to align practices with modern welfare standards.

Suggestions for Manuscript Improvement

1. Clarify sampling methodology:

- Specify exclusion/inclusion criteria (e.g., whether studies using pre-killed insects were excluded).

2. Deeper temporal analysis:

- Explore drivers behind increased sacrifice reporting (e.g., journal policy changes vs. researcher awareness).

3. Address ineffective anesthesia:

- Highlight risks of using non-veterinary-approved methods (e.g., chilling/freezing) and their scientific impacts (e.g., altered behavior in *Drosophila*).

4. Practical tools for researchers:

- Include checklists or templates for ethical reporting in supplements (e.g., ARRIVE guidelines adaptation).

5. Visual enhancements:

- Use distinct colors in figures to differentiate trends across journals or insect orders.

Notable Errors/Improvements

- Writing inconsistencies:

- Table 1 headers are unbalanced (e.g., "Papers containing sacrifice without invasive handling" could be shortened to "Sacrifice Only").

- Standardize terms (e.g., use "anesthesia" instead of "anesthetics/analgesics" in Methods).

- Referencing issues:

- Some citations include broken hyperlinks (e.g., [(Sert et al., 2020)](https://www.zotero.org/google-docs/?7BVuox)). Standardize to APA format.

- Missing figure references (e.g., "Figure 1" and "Figure 2" are cited but not included in the text).

- Language refinement:

- Avoid repetition (e.g., "increase in the proportion of papers reporting insect death" → "rising prevalence of sacrifice reporting").

Conclusion

The study provides a valuable analysis of ethical reporting gaps in insect research, emphasizing reproducibility and welfare challenges. To amplify its impact, the authors could strengthen practical recommendations (e.g., journal-policy collaborations) and refine methodological clarity. Addressing these points would position the manuscript as a cornerstone for ethical reform in entomology.

Latakia, 30/4/2025

Reviewer

Prof. Dr. Nabil ABO KAF
