# Supplementary material for: Identifying trends in reporting on the ethical treatment of insects in research
Source: PLoS One. 2025 Aug 18;20(8):e0328931. doi: 10.1371/journal.pone.0328931 (PMC12360591; doi:10.1371/journal.pone.0328931)
Supplement: Attachment — Submitted filename: R2R (1).docx [file pone.0328931.s007.docx]

**Reply to reviewers in bold**

Journal Requirements:

Please review your reference list to ensure that it is complete and correct. If you have cited papers that have been retracted, please include the rationale for doing so in the manuscript text, or remove these references and replace them with relevant current references. Any changes to the reference list should be mentioned in the rebuttal letter that accompanies your revised manuscript. If you need to cite a retracted article, indicate the article’s retracted status in the References list and also include a citation and full reference for the retraction notice.

**We don’t believe we’ve cited any retracted papers. Please let us know if that’s not the case.**

Reviewer Requests:

Reviewer #1: Thanks for the adequately addressed comments and appropriate answer for the comments. this manuscript may be helpfull fo the future study about the ethical assesments in insects

**We thank this reviewer for their comments and were glad to address them and improve the manuscript.**

Reviewer #3:

**We would like to note, for the editor’s sake and for their editorial process, that we believe the below review was generated by AI given its formatting and incorrect substance (frequently recommending the improvement of things in the manuscript already, suggesting APA formatting, etc.). We are addressing the comments anyway for due diligence, but find addressing incorrect and AI-generated reviewer comments pretty depressing.**

Suggestions for Manuscript Improvement
1. Clarify sampling methodology:
- Specify exclusion/inclusion criteria (e.g., whether studies using pre-killed insects were excluded).

**We had information about exclusion in lines 143-146. We have added a line about studies with pre-killed insects.**
2. Deeper temporal analysis:
- Explore drivers behind increased sacrifice reporting (e.g., journal policy changes vs. researcher awareness).

**We have discussed this, insofar as information is available, in lines 449-443. There are no other data to cite on policy changes or researcher awareness.**

3. Address ineffective anesthesia:
- Highlight risks of using non-veterinary-approved methods (e.g., chilling/freezing) and their scientific impacts (e.g., altered behavior in Drosophila).

**We have addressed this already in lines 369-375.**
4. Practical tools for researchers:
- Include checklists or templates for ethical reporting in supplements (e.g., ARRIVE guidelines adaptation).

**We have already pointed to the ARRIVE guidelines (line 512) and have a supplemental checklist already made in this manuscript.**
5. Visual enhancements:
- Use distinct colors in figures to differentiate trends across journals or insect orders.
Notable Errors/Improvements
- Writing inconsistencies:
- Table 1 headers are unbalanced (e.g., "Papers containing sacrifice without invasive handling" could be shortened to "Sacrifice Only").

**We will not make this change, as we want to use consistent terminology in all figures and tables.**

- Standardize terms (e.g., use "anesthesia" instead of "anesthetics/analgesics" in Methods)

**This would not be a grammatically correct change, therefore we have not made it**.

- Referencing issues:
- Some citations include broken hyperlinks (e.g., [(Sert et al., 2020)](<https://www.zotero.org/google-docs/?7BVuox)>).

**Hyperlinks will not be included in the final publication so, as long as the references are complete, we should be okay.**

Standardize to APA format.

**We have not standardized to APA as that is not the journal’s reference format.**
- Missing figure references (e.g., "Figure 1" and "Figure 2" are cited but not included in the text).

**Figures mentioned are included in the text; this is not correct.**
- Language refinement:
- Avoid repetition (e.g., "increase in the proportion of papers reporting insect death" → "rising prevalence of sacrifice reporting").

**We have opted to keep our language precise and consistent, if slightly repetitive, to ensure reader understanding of our results.**

Reviewer #4: Line 467: please correct the spelling of Lepidoptera

**We have done so.**

Line 509: I would like to see a few recommendations for proper anesthesia and euthanasia for insects. The manuscript discusses the absence of proper reporting and likely improper handling of insects in experiments, but doesn't provide options on how to improve the problem experimentally. Some entomologists may not know how to properly anesthetize insects.

**We don’t want this paper to turn into a document on how to anesthetize insects - we have already produced, and cited, other guidance in the text that entomologists looking to properly anesthetize their insects can turn to. This is important because different anesthetics, durations and dosages are recommended for different insects, so this is not a simple ‘write one sentence’ fix but rather would require an extensive amount of space be dedicated to insect anesthesia (which is valuable but not the point of the paper). We have instead revised these sentences to make it more clear that the cited references are anesthesia guides FOR insects specifically (and not animals generally): “there is also general guidance on insect anesthesia or euthanasia from veterinarians (reviewed in Barrett, 2024; and see Cooper, 2011; Wahltinez et al., 2023) and in Fischer et al. (2024) and Durosaro and Barrett (2025).”**
